# Reproductive Behaviour Evolves Rapidly When Intralocus Sexual Conflict Is Removed

**DOI:** 10.1371/journal.pone.0002187

**Published:** 2008-05-14

**Authors:** Stéphanie Bedhomme, Nagaraj G. Prasad, Pan-Pan Jiang, Adam K. Chippindale

**Affiliations:** Department of Biology, Queen's University, Kingston, Ontario, Canada; University of Exeter, United Kingdom

## Abstract

**Background:**

Intralocus sexual conflict can inhibit the evolution of each sex towards its own fitness optimum. In a previous study, we confirmed this prediction through the experimental removal of female selection pressures in *Drosophila melanogaster*, achieved by limiting the expression of all major chromosomes to males. Compared to the control populations (C_1-4_) where the genomes are exposed to selection in both sexes, the populations with male-limited genomes (ML_1-4_) showed rapid increases in male fitness, whereas the fitness of females expressing ML-evolved chromosomes decreased [1].

**Methodology/Principal Findings:**

Here we examine the behavioural phenotype underlying this sexual antagonism. We show that males expressing the ML genomes have a reduced courtship level but acquire the same number of matings. On the other hand, our data suggest that females expressing the ML genomes had reduced attractiveness, stimulating a lower rate of courtship from males. Moreover, females expressing ML genomes tend to display reduced yeast-feeding behaviour, which is probably linked to the reduction of their fecundity.

**Conclusion/Significance:**

These results suggest that reproductive behaviour is shaped by opposing selection on males and females, and that loci influencing attractiveness and foraging were polymorphic for alleles with sexually antagonistic expression patterns prior to ML selection. Hence, intralocus sexual conflict appears to play a role in the evolution of a wide range of fitness-related traits and may be a powerful mechanism for the maintenance of genetic variation in fitness.

## Introduction

The two sexes are often selected to pursue different reproductive strategies and therefore invest differently in offspring production and care, secondary sexual characters and behaviours, creating the potential for sexual conflict. The classic asymmetry is the case where females invest more in offspring production and provisioning, a resource males have to compete for. Bateman [2] characterised a male's optimal reproductive behaviour as ‘undiscriminating eagerness’ and a female's as ‘discriminating passivity’, because apparently only males benefited from promiscuity; female fitness was saturated after one or a few matings. There are notable exceptions to this situation, particularly when males largely contribute to parental care [3], [4], but it is clear that promiscuity is rampant among sexual species and that this frequently leads to different reproductive optima. This is evidenced by the widespread occurrence of sexual dimorphism, a phenomenon that reflects two genetically distinct forms of sexual conflict: conflict between different loci (interlocus) and conflict over the same locus (intralocus).

### Interlocus conflict

Interlocus conflict involves genes that are beneficial for the sex expressing them, but detrimental to the other sex, with harm being mediated through their direct reproductive interactions [5]. This creates selection pressure for the evolution, at other loci, of counteracting mechanisms in the sex experiencing harm, potentially leading to an ‘evolutionary chase’ or ‘arms race’ between the sexes [6], [7]. This evolutionary dynamic has been widely discussed and investigated in the last few years (see 8 for a thorough treatment), with no organism more studied than the fruit fly, *Drosophila melanogaster*. Recent evidence exaggerates Bateman's ‘principle’ [2], by pointing to a substantial mating cost to females. At least under some circumstances, mating multiply appears to be harmful for females [9]–[12] both as a result of behavioural interactions and as a side effect of postcopulatory sexual selection [13]–[15]. Males have evolved an array of behaviours and properties of the ejaculate to increase mating and fertilization success even when this is costly to female lifetime fitness [5] and evidence for female counter-adaptation is steadily accumulating. If promiscuity is detrimental to female fitness then a non-linear, hump-shaped – rather than plateauing – relationship between mating rate and fitness may be more realistic for females of this species [16].

### Intralocus conflict

In contrast to these scenarios involving direct interactions, intralocus sexual conflict occurs when males and females have different optima for a trait expressed in both sexes. Alleles influencing this trait are positively selected in one sex but negatively selected in the other, defining a pattern of sexually antagonistic (SA) expression. These SA alleles create a positive genetic correlation for the trait value but a negative genetic correlation for fitness between the sexes because of the opposing effects of the trait on fitness. The sexes are thus caught in a tug-of-war which impedes evolution towards their respective optima. As a result, genes maladapted to the sex they are expressed in create what has been called a ‘gender load’ on the population [1], [17]. Intralocus conflict can be resolved by mechanisms such as sex-limited expression of SA loci, translocation of SA genes to the sex chromosomes [18], [19] and genomic imprinting [20]. Such resolution is manifested through sexual dimorphism, a widespread feature among sexually reproducing species [21].

Despite these remedies, evidence of the persistence of sexually antagonistic variation has recently accumulated in organisms as diverse as snakes [22], lizards [23], crickets [24], dioeceous plants [25], sheep [26], red deer [27] and perhaps even humans [28]. As with interlocus conflict, the clearest evidence of intralocus sexual conflict has been established in *Drosophila melanogaster* where the exact same “hemiclones” (haplotypes consisting of cloned copies of all major chromosomes in the *D. melanogaster* genome) have been expressed in males and females, revealing a negative genetic correlation for adult fitness between the sexes [17], [29], [30]. While these studies unambiguously established a pattern of sexual antagonism, they had some limitations from a quantitative perspective. For example, relative fitness is sensitive to the distribution of genetic variation sampled. Moreover, the combination of positive (e.g., because of mutation selection balance) and negative genetic correlations between the sexes at different loci frozen within a hemiclone may obscure the effects of sexually antagonistic genes.

The result of mixed positive and negative correlations was illustrated in total fitness in the Chippindale *et al.* study [29]. Total (or net) fitness is the product of survival, which was positively correlated between the sexes, and fertility, which was negatively correlated, producing a pattern in which fitness was uncorrelated between the sexes. Such a pattern is indistinguishable from sex-limited gene expression, and while that would also point to a history of divergent selection for separate fitness optima, it would reflect a fundamentally different pool of standing genetic variation. Even if fitness is the ultimate currency for measuring conflict, to get a complete picture and avoid the problem of intersexual genetic correlations of mixed sign, we need to characterize the phenotype in more detail. These earlier studies only measured juvenile viability and adult fertility and did not identify any specific traits underlying the intralocus conflict.

### Using male-limited evolution to study the evolutionary implications of sexual conflict

In order to further explore the genetic and phenotypic nature of sexual conflict, we have recently used an experimental evolution approach [1]. Experimental evolution improves on single-generation assessments of quantitative genetic variation by (1) sampling a much greater number of initial genotypes, (2) allowing recombination and selection to concentrate adaptive characters within the focal populations, by (3) unfreezing the artificial linkage established in randomly sampled genotypes, reducing the problem of detection of SA variation and (4) facilitating the measurement and analysis of relevant phenotypes. We used the ingenious male-limited evolution procedure developed by W.R. Rice [11] to eliminate sexual conflict. In this procedure, hemiclones (intact haploid genomes) are transmitted from father to son only, using specialized genetic constructs. Theoretically, this patrilinear transmission of all major chromosomes will completely remove female-specific selection pressures.

By removing counter-selection in females, we expected to see sexually antagonistic male-benefit traits evolve because of the release of both inter- and intralocus sexual conflict. The first kind of conflict was the main focus of Rice's work [11] although the problem of intralocus conflict was not completely overlooked in this experiment [31]. During the maintenance of these lines, all grandmaternal chromosomes are discarded, and the females used to propagate male-limited haplotypes are derived *de novo* each generation from a separate stock. Therefore, males have the opportunity to adapt to clone-generator females, but these females do not have the potential to counteradapt to males. Rice showed extensive evidence for the rapid adaptation of these males to the females, implying that male-limited evolution had given males the upper hand in the arms race between the sexes.

We mainly focussed on the other part of the story: If genetic variation for fitness is maintained in populations due to intralocus sexual conflict, then release from selection on female function should lead to increased male fitness, as noted above, but also a corresponding decline in the fitness of females expressing male-evolved genes. This is the pattern documented by Prasad *et al.* [1]. There we showed that after 25 generations of experimental removal of females from the gene pool, male fitness had increased by 15% and that the fitness of females expressing male-limited evolved genomes was reduced by 10%. We also documented changes in size, development time and growth rate in both sexes that were consistently in the male direction of evolved sexual dimorphism, suggesting a masculinization of the developmental program. These results reveal that intralocus sexual conflict genes were polymorphic in the ancestral populations and probably play a significant role in the maintenance of genetic variation in fitness. Moreover, they point out the value of analyzing a more detailed phenotype: SA variation was present in the juvenile stages but it was not related to viability, the character previously used as a measure of juvenile fitness [29], [30].

### Reproductive behaviour and sexual conflict

Behaviour associated with reproduction is likely to be important in the evolution of sexual conflict and is the main focus of the present study. Courtship involves a variety of visual, acoustic and chemical signals [32], and given the cost of mating in *D. melanogaster* is likely to be an arena for interlocus sexual conflict. However, this complex physical and ethological phenotype may also vary as a result of intralocus sexual conflict if signalling characters are shared by the two sexes. The visual signals in *D. melanogaster* courtship mainly consist of courtship dances, wing displays, while wing vibrations that produce an acoustic “love song” also play an important role in both species recognition and mate choice [33]. Cuticular hydrocarbons, some of which also serve as pheromones in *D. melanogaster* and related species, are chemical signals involved in species and sex recognition [34]–[36], in stimulating male courtship [37] and in mate choice [38]. The combination of these signals and the receptivity of the female determine the ‘efficiency’ of the courtship. Courtship, and some of its components, have been shown to possess substantial quantitative genetic variation in *D. melanogaster* [39], [40], indicating that they have evolutionary potential. Among the different signals exchanged, some are one-way signals (e.g., the male acoustic signal) whereas others are shared but sexually dimorphic signals (e.g. pheromones and visual signals). These asymmetrical conjointly expressed traits are potential candidates for intralocus sexual conflict. For example, males inheriting more ‘feminine’ patterns of cuticular hydrocarbon expression may have lower reproductive success.

Other behaviours potentially involved in intralocus sexual conflict are foraging and mate-seeking. In *D. melanogaster*, as in many insects, female fecundity and protein acquisition are closely linked. In laboratory *Drosophila*, this typically results in a tight correlation between yeast consumption and fecundity [41], [42]. In contrast, male fitness is not strongly influenced by yeast consumption [43] and males do not feed detectably on supplemental live yeast early in life [42]. At least in short-generation fly stocks, males appear to forego feeding opportunities in favour of mate-seeking (or other) behaviours that are related to fitness. Hence, if a trade-off exists between feeding and mate-seeking or courtship-related activity, it might be an important arena for sexual conflict. A female inheriting a more ‘masculine’ pattern of activity may suffer reduced fitness by losing out in competition for fecundity-limiting resources or through expensive and ineffectual behaviours. This appears to be the mechanism underlying the intersexual tradeoff in locomotor activity recently documented in *D. melanogaster* [44]. Using hemiclone analysis, this study shows that high locomotor activity is correlated with higher fitness in male flies, but female fitness is reduced by expression of high activity genotypes.

In the present study, we extend analysis of male-limited evolved populations to behavioural characters involved in courtship, mating and feeding activity. The role of these traits in sexual conflict has never been analysed using an experimental evolution approach. Our hypothesis is that gender specificity of optimal behaviour underlies part of the sexually antagonistic selection response observed under male-limited evolution. If so, removal of selection for behavioural traits related to female fitness (e.g., yeast foraging) should lead to more masculine patterns of behaviour in both sexes when they express male-limited evolved genomes, with opposite effects on fitness.

## Results

### Accounting for changes in mating status

Observations of courtship and mating activity were made beginning almost immediately after combining the males with the virgin females in vials. This protocol was designed to measure male courtship effort and realized success longitudinally, with both virgin females and mated females, where the latter are expected to be more discriminating. These data can also be used to calculate a measure of ‘courtship efficiency’: the number of courtship events required for a male to secure a mating. However, an examination of the temporal distribution of the data indicated that a large proportion of the observed copulations were made during the first observation round, corresponding to virgin matings. Previous experiments [11], [45] confirmed that virtually every sexually mature female from the LH population will complete a first copulation in a span of less than 2 hours if put in the presence of males. Because females were in two different reproductive states at the beginning and end of the observation period, we examined the data both globally and using data from just the third to the seventh observation rounds, corresponding to 2 h15 after combination of experimental animals and consequently to non-virgin matings.

### Male behaviour

**Figure 1:**
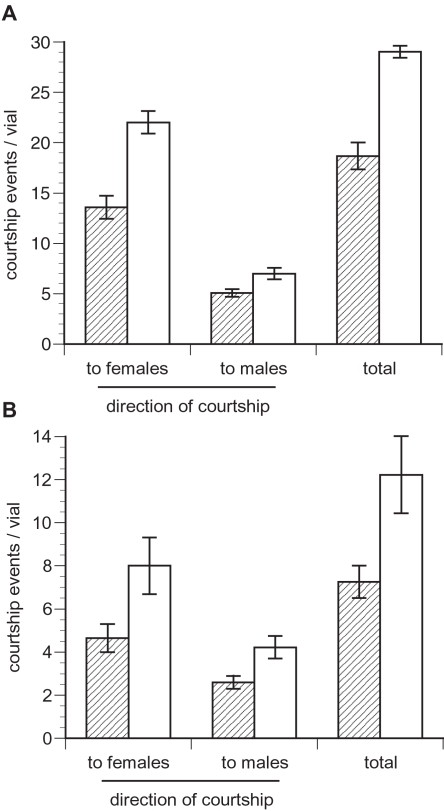
Total number of courtship events and their orientation observed in 7 hourly checks of each vial (± s.e.) when male-limited (ML) evolved chromosomes (shaded bars) and Control (open bars) were expressed in males (A) and in females (B). All measurements are from vials housing both sexes. Males expressing ML chromosomes courted at a significantly lower rate than control males did. Females expressing ML chromosomes were courted at a significantly lower rate than controls, and were also associated with lower rates of homosexual courtship.

**Figure 2:**
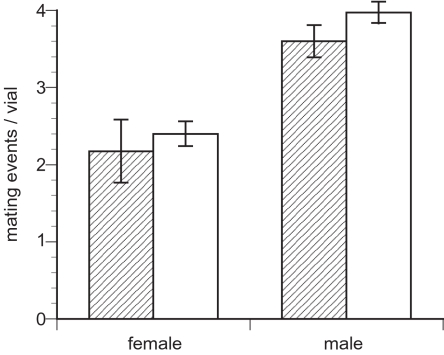
Total number of matings observed (± s.e.) per four pairs in a vial when male-limited evolved chromosomes (shaded bars) and C (open bars) are expressed in females (left side) and in males (right side). No significant differences in mating rate were detected between selection treatments in either sex. Because of the spot check strategy employed (checks every hour, with mating lasting 17 minutes, on average), mating rate estimates are several times lower than real mating rates.

In order to determine if intralocus sexual conflict affected the evolution of courtship intensity and mating frequency, we recorded courtship and matings of males expressing ML and C genomes. Over the whole observation period, males expressing ML-evolved genomes showed lower levels of courtship activity compared to control males (t_3_ = 8.21, p = 0.004, figure 1A). This reflected both a decrease in courtship directed to females (t_3_ = 6.18, p = 0.009, figure 1A) and to other males (t_3_ = 3.45, p = 0.041, figure 1A, table 1). Nonetheless ML males acquired matings at the same rate as did C males (t_3_ = 1.22, p = 0.309, figure 2, table 1). As noted above, however, if matings with less-discriminating virgin females accounted for the majority of our observations, then this result could be interpreted as a generic reduction in male mating effort; an unexpected result given increased competitive fitness in the ML lines. Because of the observation protocol, the number of matings actually occurring could be as much as 3 times the number observed; we checked the vials hourly but the mounted period in *D. melanogaster* lasts for approximately 15 to 20 min (16.5 ± S.D. 3.3 minutes in the LH_M_ population; J. Sy, pers. comm.).

**Table 1:** Courtship, mating and feeding data for observations during the virgin and non-virgin matings combined (observation points 1 to 7).

		Number of courtship events (s.e.)			Number of matings (s.e.)	Number of occurrences at the food source (s.e.)
		Toward males	Toward females	Total		
Males	Male-limited	5.08 (0.36)	13.6 (1.14)	18.68 (1.34)	3.60 (0.21)	10.68 (0.40)
	Control	7.00 (0.58)	22.03 (1.13)	29.03 (0.59)	3.98 (0.14)	10.38 (0.88)
Females	Male-limited	2.60 (0.30)	4.65 (0.66)	7.25 (0.75)	2.18 (0.41)	9.2 (0.97)
	Control	4.23 (0.52)	8.00 (1.31)	12.23 (1.78)	2.4 (0.16)	11.98 (0.79)

See Methods for details.

We separately examined the number of matings and courtship events observed from the third to the seventh observation rounds, when virtually all females were expected to have mated at least once. During this interval, males expressing ML-evolved genomes showed lower levels of courtship activity compared to control males (t_3_ = 7.65, p<0.01). This split into a significant decrease in courtship directed to females (t_3_ = 5.54, p = 0.01) and a marginally non-significant decrease in courtship directed to other males (t_3_ = 3.07, p = 0.06, table 2). Nonetheless ML males acquired matings at the same rate as did C males (t_3_ = 0.83, p = 0.47, table 2).

**Table 2:** Courtship, mating and feeding data for observations during the virgin and non-virgin matings combined (observation points 3 to 7).

		Number of courtship events (s.e.)			Number of matings (s.e.)	Number of occurrences at the food source (s.e.)
		Toward males	Toward females	Total		
Males	Male-limited	4.28 (0.23)	12.55 (0.98)	16.83 (1.14)	0.90 (0.15)	9.13 (0.18)
	Control	5.65 (0.46)	20.30 (1.31)	25.95 (0.89)	1.10 (0.10)	8.68 (0.36)
Females	Male-limited	2.33 (0.28)	3.25 (0.39)	5.58 (0.59)	0.30 (0.14)	3.98 (0.63)
	Control	3.53 (0.43)	5.90 (0.94)	9.43 (1.35)	0.28 (0.08)	6.38 (0.93)

See methods for details.

**Figure 3:**
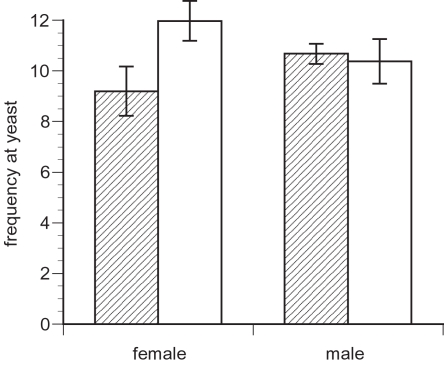
Number of individuals present at the yeast food source (± s.e.) when ML chromosomes (shaded bars) and C chromosomes (open bars) were expressed in females (left side) and males (right side) in vials containing individuals of both sexes.

We also measured the time spent at the yeast food source, another trait identified as a potential sexually antagonistic trait. For this trait, we found no difference between ML and C males, either over the whole experiment (t_3_ = 0.33, p = 0.76, figure 3, table 1) or in observation periods three to seven (t_3_ = 1.28, p = 0.29, table 2). This absence of a difference in time spent at the yeast food source was confirmed in the “male only” vials (t_3_ = 0.95, p = 0.41 for the full observation time and t_3_ = 0.61, p = 0.59 for the observation rounds 3 to 7). In these vials, we also recorded the male-male courtship activity and found a reduced male-male courtship activity of ML males compared to the C males (t_3_ = 3.45, p = 0.04).

### Female behaviour

As some of the signals exchanged during courtship are bidirectional and for this reason potentially sexually antagonistic, we were interested in the reaction of unrelated tester males in the presence of females expressing the male-limited genomes. We measured the level of courtship directed towards ML and C females as well as the level of male-male courtship in their presence. As for the male observations, we present data for the full observation period (corresponding to both virgin and non-virgin matings) and for observation points 3 to 7 separately (non-virgin females). Over the full observation span, the global level of courtship activity (courtship directed to males and females) was lower in vials containing ML-expressing females than in vials containing control females (t_3_ = 3.48, p<0.05, figure 1B, table 1). This decrease split into a marginally non-significant decrease of male courtship activity directed to females (t_3_ = 2.78, p = 0.07, figure 1B, table 1) and a significant decrease in courtship directed to other males (t_3_ = 7.00, p<0.01, figure 1B, table 1). The same analysis performed for the observation rounds 3 to 7 indicated that the courtship activity was lower in vials containing ML females than in those containing control females (t_3_ = 5.02, p<0.05, table 2). This split into a significant decrease in courtship directed to females (t_3_ = 4.65, p<0.05, table 2) and to males (t_3_ = 5.76, p = 0.01, table 2). However, the total number of matings was not different for females expressing the ML and the C genomes either for the full observation time (t_3_ = 0.45, p = 0.68, figure 2, table 1) or for non-virgin matings (t_3_ = −0.12, p = 0.91, table 2).

We measured the time spent at the food source (a point source of concentrated yeast in the vial), to look for a trade-off between feeding frequency and courtship-related activity. For this trait, there was a non-significant trend, with ML-expressing females spending about 15% less time oriented towards the yeast than the C females (t_3_ = 2.72, p = 0.07, figure 3, table 1). Similarly, in the “female only” vials, ML females tended to feed less than control females (t_3_ = 2.59, p = 0.08). Females didn't show any recognizable courtship activity towards each other.

## Discussion

The present experiments confirm the hypothesis that behavioural characters related to reproductive success evolve rapidly when one sex is removed from the gene pool. And, for the most part, our findings, combined with our previous results on the fitness evolution of these lines, are interpretable within the framework of intralocus sexual conflict; consistent with a masculinization of the genome under exclusive male selection pressures. Our results suggest that gains in male fitness [1], were accompanied by a decrease in the courtship rate of males, while females experimentally expressing these ML genomes were less courted by males and tended to show reduced foraging rates, despite the critical relationship between protein-feeding and fecundity. The reduction in male courtship effort by ML-expressing males was not predicted and deserves careful consideration in light of the experimental design and the species' biology. However, this decrease in courtship activity did not lead to a decrease in the number of copulations observed, and we know from other experimental assays [1] that these males have higher fitness than control males do. Below we discuss potential reasons for reduced courtship frequency by ML males, both adaptive and artifactual.

### Why Reduced Courtship Rate?

One of our main results was that ML-evolved males courted substantially less than controls (e.g., 38% less with non-virgin females). This result was surprising because these populations had increased in male fitness and we expected elevated rates of courtship towards females to be part of that response. There are four distinct explanations for this result and a variety of additional points for discussion that follow. The first is that ML males were more efficient at courting, and that in a more competitive mating environment, or over an extended time frame, their behaviour would pay off in greater total mating success. The second is that the ML populations had declined in vigour since last being assayed and this was reflected in reduced courtship effort; we observed equivalent mating rate because of the relatively indiscriminate mating habits of virgin females. Third, our behavioural observations, based on frequency of observed courtship, might not capture investment in the intensity or quality of courtship. And fourth, differences between the experimental assay environment and the respective selection regimens led to reduced courtship by ML-evolved males as an artefact.

In order to untangle the first two alternatives, it has to be noted first off that the decline in courtship rate under ML selection was not associated with a decline in the number of matings acquired throughout the observation period. Our experiment was initiated with sexually mature virgin females, who mate readily when combined with males. In fact, using the present protocol in earlier experiments, we have reliably observed nearly 100% of females mating within the first hour of being combined with males. If we had scored predominantly virgin matings, then it would be unsurprising to see no differences in total mating success, even with fairly substantial differences in male virility or attractiveness. Under this scenario, mating would have been concentrated at the beginning of the observation period, and the per female copulation rate estimate would be close to one. The protocol, involving hourly checks, is certain to have missed copulations that occurred between checkpoints because amplexus occurs for 15-20 minutes, on average, in our lab stocks (unpublished data). Hence the direct observation of 3.3 copulations per group of four females (0.88 matings/female) over the course of the experiment suggests that two to three copulations per female actually occurred. We also looked specifically at mating and courtship frequency from three hours on, when virtually all females would be carrying sperm and observed lower courtship rates and equivalent mating success by the ML males under these conditions. Finally, although we did not actually measure male fitness at the same generation as we observed the reproductive behaviour (generation 52), a fitness advantage for ML males was present both at generation 25 [1] and 75 (unpublished data). Consequently, we assume that the fitness of ML-expressing males was higher when the present study was performed.

We measured the courtship activity by checking each vial once per hour and counting courting pairs. This measure does not allow us to evaluate the intensity or quality of courtship. It may be that the ML males evolved a more vigorous courtship ritual and invested as much energy in courtship as the C males through shorter but more intense courtship displays. Whatever the precise nature of the behavioural change, our recorded decrease in courtship activity revealed an evolved difference between ML and C males and a likely change in courtship strategy when female selection pressures are removed.

The fourth hypothesis for reduced courtship frequency by ML-selected males is differences between the evolutionary treatments of the 2 types of populations and the conditions in which we observed their behaviour. Despite our efforts to closely match evolutionary and experimental conditions, a few differences were inevitable. First ML and C males have evolved with different types of females: ML males normally mate with “clone generator females”, which carry a bevy of markers that make them unusual (i.e., yellow-bodied, orange-eyed, forked-bristled). Yellow, for example, is well-known to affect sexual selection when males carry it [46], and under this protocol males may have evolved a preference for this “Frankenfly” female phenotype. Clone-generator females are also much frailer than the virtually wildtype LH_ST_ females used in these experiments, so the reduction in courtship intensity could have evolved to reduce harm to females, although this speculation is inconsistent with Rice's findings [11]. The important point here is that the LH_st_ females used in courtship experiments were genetically and phenotypically more similar to the wildtype females used in maintaining the control populations and relatively foreign to the ML males. Another difference between selection and experimental assay was that we observed courtship and mating behaviour for only 7 h and only in daylight, whereas during the regular selection protocol, males and females are allowed to interact for 18 h, including day (6 h) and night (12 h) light regimes. By observing only a portion of the interaction time, we may have missed differences attributable to night-specific courting behaviour or that only matter later on in the normal interaction period. In other words, it is possible that we did not observe a higher number of mating because our window of observation did not cover a period as long as the interaction period during the regular selection protocol or include night time observations. It also has to be noted that there was a methodological difference between fitness assays and the present study: fitness was evaluated with focal males (ML or C) put in competition with standard males whereas here, each type of male was alone with the females. Thus it might be that the different courtship behaviour of ML males only translates into a higher number of matings when the males are in competition with, or compared to, standard males.

### Male attractiveness

The reasons for reduced courtship rates of the ML line males cannot be resolved here, but our present results and competitive fitness [1] data nonetheless suggest that tester females found them more attractive than control males, accepting similar numbers of mating from ML males despite their reduced duration of courtship. This increased attractiveness may be related to various characteristics, including quality or quantity of epicuticular pheromones and more effective courtship dance or “love song”.

ML males have been shown to be slightly smaller than control males [1]. In that study, we noted that growth, size and development had all shifted in the ‘male direction’ of ancestral sexual dimorphism and suggested that this reflected masculinization of the phenotype associated with higher fitness. Female-biased sexual size dimorphism is often attributed to fertility selection, but, in *D. melanogaster*, males are not only smaller, but later-emerging and substantially slower growing than females are. Small size could give an advantage to males during courtship because being smaller allows them to be more agile. Smaller size is thought to give an agility advantage in flying species, particularly during courtship, including *D. subobscura* [47], midges [48] and bustards [49]. Moreover, smaller size and slower growth rate may promote a more developmentally stable individual if ontogenetic fidelity is costly in terms of growth [1], [50], [51]. In other words, ML-evolved males may be more attractive because they are in some way better formed for male function due to slow growth. Symmetry is one candidate that has been related to higher attractiveness in several species [52], including *D. melanogaster* [53] and *D. bipectinata* [54] although counter-results exist [55]. Polak and Taylor [54] show the positive size-scaling of fluctuating asymmetry that would be required to promote smaller size in the ML lines. This hypothesis remains to be verified by morphometric analysis of the ML and C lines, which is currently underway. At the very least, the evolution of smaller size and higher fitness in our ML selection experiment, in the absence of differential demographic, density, or developmental selection is suggestive of a link between aspects of the sexual phenotype and the removal of selection for female function.

### Homosexuality

In *D. melanogaster*, males engage in fairly high levels of same sex courtship [56]. If homosexual behaviour is a costly byproduct of selection on some aspect of female behaviour or mate-recognition, then we should expect it to decline under ML-selection. For example, genetic factors increasing female fitness and promoting male homosexuality have recently been suggested in humans [28]. We did see lower courtship activity directed towards other males by ML males. However, there was no significant difference in the proportion of courtship directed to males (t_3_ = −1.48, p = 0.35) leaving open the possibility that reduced same-sex courtship was a pleiotropic effect of reduced courtship activity towards females, or part of a general syndrome of reduced activity.

### Experimentally-Produced Daughters are Less Attractive

ML-evolved chromosome expression by females resulted in reduced courtship rates of tester males towards them. This can either be due to the fact that they had evolved to be less attractive to males or that they evolved a lower resistance to male harassment so that males have to court them less to gain the same number of matings. These two potential explanations are actually not exclusive and can both be a signature of the masculinisation of the ML genomes. Indeed, the lower courtship activity detected in these circumstances may be due to the ‘masculinisation’ of a variety of characteristics relating to female attractiveness. For example, we already know that ML-expressing females are smaller than control females [1] and there is some evidence that males prefer to mate with large females [57]. ML-expressing females may also have more “male-like” pheromones and for this reason exert weakened stimulation. Female pheromones have been shown to have a stimulatory effect on courtship, including on courtship directed to males [37], supporting the idea that pheromones play a role in the observed behavioural changes. Along these lines, we also found a decrease in the rate of male-male courtship in the presence of ML-expressing females. This result again suggests that male homosexual interactions may be triggered and modulated by their level of excitement towards females.

### Experimentally-Produced Daughters Forage Less

We predicted that feeding behaviour would decline under ML evolution if there were a trade-off between feeding and other activities such as courtship, territoriality or locomotor activity associated with male fitness. Just such an intersexual trade-off for locomotor performance has recently been described in the ancestor population to the ML and C populations (LH_M_) using hemiclone analysis [44]. Whereas female fitness is closely associated with early yeast feeding, as discussed above, males consume live yeast at an undetectable rate early in life. Hence, a male's fitness may be compromised if he inherits female-selected alleles related to reduced overall activity, reduced aggression, or increased foraging behaviour. The results we obtained go in the direction of this prediction: When females expressed ML genomes they tend to spend less time in contact with the yeast supply (p = 0.07 for mixed vials and p = 0.08 for female-only vials). Whether reduced yeast feeding causes reduced fecundity, or the other way around, and what other behaviours replaced feeding could not be deduced from these data. We merely report that females spend less time oriented towards a concentrated point source of food when they inherit masculinized genomes.

On the other hand, male ‘feeding behaviour’ was not affected by selection history. Lack of change in male behaviour is perhaps unsurprising. We know from our own unpublished assays, as well as Stewart *et al.* [42] from the same system, that males in these populations do not feed measurably early in life; their occurrence at the yeast point source may be coincidental to other activity. If selection has been for, say, higher locomotor activity in the ML-populations, then females expressing ML-genomes are less likely to sit and feed at the yeast source, providing a mechanism for the trade-off observed by Long and Rice [44].

An alternative explanation for reduced female feeding (and fitness in general) is mutation accumulation in female-expressed genes, sheltered from selection in the ML populations. For example, a degradation of female-specific up-regulation of feeding activity may have occurred under relaxed selection for female behaviour. Changes in the mutation-selection balance for sex-limited genes are part of such a selection protocol, but, given the small proportion of sex-limited loci in the *D. melanogaster* genome [58] and the low rate of mutation accumulation expected in fairly large populations such as these [59], they are unlikely to explain a major fraction of the fitness changes observed in the ML lines.

### Summary

Male-limited evolution has led to increases in fitness that we have now linked to shifts in developmental characters, size, and adult behavioural phenotypes towards the male-specific optima suggested by sexual dimorphism. In other words, selection for male-specific function has masculinized the allelic composition of the genome or the level of gene expression of dose-dependent genes. This interpretation (rather than adaptation to the genetic constructs or environmental conditions used in ML evolution) is confirmed by the observation that when females express male-evolved genomes, their fitness is sharply reduced and they are also phenotypically more male-like [1].

Although we are cautious in our evaluation of the courtship data, it seems likely that ML males had higher fitness when in competition with other males because they were more attractive to females. Changes in behaviour, size and other morphometric characters are potentially linked to the sexually antagonistic selection response. Other sexual signals, such as cuticular hydrocarbon (CHC) pheromones, are promising candidates for sexually antagonistic effects. CHC profiles are markedly sexually dimorphic in terms of the relative investment in long-chain hydrocarbons in *D. melanogaster* [60], and they also exhibit considerable genetic variation [61].

Our results add to a growing list of studies suggesting that intralocus sexual conflict is an important agent promoting the maintenance of genetic variation for fitness. A negative intersexual correlation for fitness has profound implications for models of sexual selection via ‘good genes’ benefits. If fitness reverses itself across the sexes, then the benefits of sexual selection will either be reduced or completely short-circuited, as recently highlighted in empirical work [27], [62] and theoretically [63]. This pattern of heredity, particularly when alleles are associated with sex chromosomes, will promote the maintenance of genetic variation for fitness, even in the face of strong sexual selection [30], [64].

## Materials and Methods

### Derivation of the male-limited lines

The derivation of the male-limited (ML) lines and their matching controls (C) is described in detail in [1]. Briefly, 4 large subpopulations were derived from a long-term outbred population (LH_M_ ; described in [45]) and maintained isolated for 10 generations. From each of these populations, one pair of selected (ML_1- 4_) and control (C_1-4_) populations was initiated. Each selected and control population bearing the same numerical subscript were more closely related to each other through common ancestry and subsequent handling than to other selected or control populations. To initiate a ML population, 1040 whole-genome hemiclones, consisting of cI (X), cII, and cIII, but not the tiny dot cIV (about 99.5% of the fly genome in total) were sampled using “clone generator females”, carrying a compound X(C(1)DX, *y, f*), a Y chromosome (from LH_M_ base population) and a homozygous-viable translocation of the two major autosomes (T(2∶3)*rdgc st in ri p^p^ bw*). These chromosomal constructs and the absence of molecular recombination in male *D. melanogaster* mediate the transmission of the cI,II,III hemiclone (hereafter referred to as hemiclones) from father to son. The males carrying a translocation and a hemiclone originally sampled from LH_M_ were crossed each generation to “clone generator females”. In this way, these hemiclones were transmitted from father to son only, the grand-maternal hemiclone being dumped every generation. Efforts were made to standardize the effective population size between selected and control populations by maintaining the same number of haploid genomes in each, and the exact same maintenance protocol was used for C and ML populations, except that the C populations had normal transmission of genetic material from one generation to the next, via both males and females.

This experimental protocol completely prevented recombination in the ML populations, which could slow down their rate of adaptation due to hitchhiking and background selection. To prevent this, each generation, 4% of the genomes were passaged through a series of crosses, in which the ML hemiclones were expressed in a female for one generation, allowing them to recombine [1]. These recombined ML hemiclones were then reintroduced into the general ML population from which they were drawn.

All flies were raised on molasses-cornmeal-yeast medium at 25°C and 50% relative humidity in a 12∶12 h light/dark cycle under moderate densities of approximately 150 larvae per vial. These general conditions were identical to the ancestor LH_M_ population, however because of the complexity of the ML protocol, changes were made in the handling of adults, such that males were crossed (in both C and ML treatments) to virgin females and allowed to interact for 18 h prior to female oviposition.

### Expression of male-limited and control genomes in males and females

a. Expression of ML genomes in males.

Males used in assays had an entire ML haploid genome paired with a random ancestral genetic background. To produce these males, two consecutive crosses were necessary:

Cross 1: At generation 52 of experimental evolution, 150 haploid genomes per population (ML_1-4_ and C_1-4_) were captured by crossing males with clone generator females bearing a dominant eye-colour marker (*bw^D^*).

Cross 2: F1 progeny males were mated to females carrying a compound X(C(1)DX, *y, f*), a Y chromosome (from LH_M_ base population) and 2 sets of LH autosomes.

Red-eyed male progeny from this cross expressed the genomes of interest, while brown-eyed males and any females were discarded.

b. Expression of ML genomes in females.

Females used in assays expressed two X chromosomes from the ML population, while a set of ML autosomes was paired with a set of autosomes from the ancestral (LH_M_) population. The production of females expressing the ML genomes initially followed the same protocol (crosses 1 and 2) except that the hemiclones were sampled at generation 47. The brown-eyed male progeny produced by cross 2 were then mated to females homozygous for a balancer X chromosome (FM7). This cross produced females with a balanced ML X chromosome in an otherwise random background. These females were then crossed again to F1 brown-eyed male progeny. Red-eyed females from this cross expressed the genome of interest in a wildtype, outbred state. Through these crosses 150 haplotypes and 150 X chromosomes were sampled from the ML population. Consequently, the probability that an experimental female carries 2 copies of the same X, and suffer from inbreeding, is very low (1/22,500 if we assume random matings and equal reproductive success).

c. Production of control males and females.

For both males and females, the matching controls were obtained through exactly the same crosses but started with C males in the cross 1.

### Courtship observations for male and female assays

Males expressing the ML or C genomes were collected 12 days of age (from the day eggs were laid) under light CO_2_ anesthesia. Day 12 was sufficiently late to allow us to collect from the entire emergence time distribution (and consequently the entire body size distribution) for both ML and C populations, even if ML have a longer developmental time than C. Males were placed in observation vials in two conditions – males only (8 males per vial, 10 vials per population) and “male and female” vials (4 pairs per vial, 10 vials per population). In the “males and females” vials, the females were from a marked population expressing the relatively benign recessive scarlet-eyed mutation (LH_st_) in the LH_M_ population background. These females were collected as virgins and transferred to yeast-supplemented food vials on day 12. Flies were transferred to observation vials on day 13, just before observations started and males and females were combined at the same time for the “males and females” vials. Observation vials contained molasses-cornmeal-yeast medium, with 5 mg of yeast. This controlled for behavioural differences due to resource variation, while also mimicking selection conditions.

Very similar settings were employed for the observation of females expressing the ML genomes and their matching controls. Virgin females were collected within 6 h of eclosion and transferred to yeasted vials day 12. On day 13, females were placed in clear observation vials in two conditions – female only (8 females per vial, 10 vials per population) and “male and female” vials (4 pairs per vial, 10 vials per population). In the “males and females” vials, the males were taken from the LH_st_ population. Observation vials were identical to those used for males.

The densities in observation vials constituted a compromise between easy and accurate observation conditions and social conditions similar to the regular maintenance of the selected and control populations, where 20 males interact with 15 females. The timing of collection, transfer on yeasted vials and combination of males and females also mimics the regular maintenance of these populations.

During the two assays (male expression and female expression), vials were randomized in observation boxes and placed in a room at 25°C and bright overhead lighting. Observers were blind to the type of flies they were observing. On day 13, each vial was observed hourly for 30 seconds. The first observation round started at 10:00 a.m. and occurred exactly 15 minutes after combination for each vial. 6 other observation rounds followed. Courtship events, directed to females or to males, were recorded. One courtship bout was defined as the combination of circling around or following the target flies and displaying wings. Successful matings were also recorded. At the beginning of each round of observation, the number and the sex of flies with their heads in contact with the point-source of yeast, apparently feeding, was recorded.

### Statistical analyses

Data were submitted to paired t-tests, since ML and control populations were paired by ancestry. The replication level was the population. p-values given in the result section correspond to a two-tailed test. All analyses were implemented using JMP statistical software (version 5.0.1a).

## References

[1] Prasad NG, Bedhomme S, Day T, Chippindale AK (2007). An Evolutionary Cost of Separate Genders Revealed by Male-Limited Evolution.. Am Nat.

[2] Bateman AJ (1948). Intra-sexual selection in Drosophila.. Heredity.

[3] Trivers RL, Campbell B (1972). Paternal investment and sexual selection.. Sexual selection and the descent of man.

[4] Jones AG, Walker D, Avise JC (2001). Genetic evidence for extreme polyandry and extraordinary sex-role reversal in a pipefish.. P Roy Soc Lond B.

[5] Chapman T, Arnqvist G, Bangham J, Rowe L (2003). Sexual conflict.. Trends Ecol Evol.

[6] Parker GA, Blum MS, Blum NA (1979). Sexual selection and sexual conflict. Sexual Selection and Reproductive Competition in Insects.

[7] Rice WR, Holland B (1997). The enemies within: intergenomic conflict, interlocus contest evolution (ICE), and the intraspecific Red Queen.. Behav Ecol Sociobiol.

[8] Arnqvist G, Rowe L (2005). Sexual conflict..

[9] Fowler K, Partridge L (1989). A cost of mating in fruit flies.. Nature.

[10] Chapman T, Trevitt S, Partridge L (1994). Remating and male-derived nutrients in *Drosophila melanogaster.*. J Evol Biol.

[11] Rice WR (1996). Sexually antagonistic male adaptation triggered by experimental arrest of female evolution.. Nature.

[12] Pitnick S, Garcia-Gonzalez F (2002). Harm to females increases with male body size in *Drosophila melanogaster.*. P Roy Soc Lond B.

[13] Wigby S, Chapman T (2005). Sex peptide causes mating costs in female *Drosophila melanogaster.*. Curr Biol.

[14] Lew TA, Morrow EH, Rice WR (2006). Standing genetic variance for female resistance to harm from males and its relationship to intralocus sexual conflict.. Evolution.

[15] Linder JE, Rice WR (2005). Natural selection and genetic variation for female resistance to harm from males.. J Evol Biol.

[16] Friberg U, Arnqvist G (2003). Fitness effects of female mate choice: preferred males are detrimental for *Drosophila melanogaster* females.. J Evol Biol.

[17] Rice WR, Chippindale AK (2001). Intersexual ontogenic conflict.. J Evol Biol.

[18] Rice WR (1987). Genetic hitchhiking and the evolution of reduced genetic activity of the Y-sex chromosome.. Genetics.

[19] Rice WR (1994). Degeneration of a non recombining chromosome.. Science.

[20] Day T, Bonduriansky R (2004). Intralocus sexual conflict can drive the evolution of genomic imprinting.. Genetics.

[21] Bedhomme S, Chippindale AK, Fairbairn DJ, Blanckenhorn WU, Székely T (2007). Irreconcilable differences: When sexual dimorphism fails to resolve sexual conflict.. Sex, Size and Gender Roles: Evolutionary studies of sexual size dimorphism.

[22] Forsman A (1995). Opposing fitness consequences of colour pattern in male and female snakes.. J Evol Biol.

[23] Calsbeek R, Sinervo B (2004). Within-clutch variation in offspring sex determined by differences in sire body size: cryptic mate choice in the wild.. J Evol Biol.

[24] Fedorka KM, Mousseau TA (2004). Female mating bias results in conflicting sex-specific offspring fitness.. Nature.

[25] Delph LF, Gehring JL, Frey FM, Arntz AM, Levri M (2004). Genetic constraints on floral evolution in a sexually dimorphic plant revealed by artificial selection.. Evolution.

[26] Robinson MR, Pilkington JG, Clutton-Brock TH, Pemberton JM, Kruuk LEB (2006). Live fast, die young: Trade-offs between fitness components and sexually antagonistic selection on weaponry in Soay sheep.. Evolution.

[27] Foerster K, Coulson T, Sheldon BC, Pemberton JM, Clutton-Brock TH (2007). Sexually antagonistic genetic variation for fitness in red deer.. Nature.

[28] Camperio-Ciani A, Corna F, Capiluppi C (2004). Evidence for maternally inherited factors favouring male homosexuality and promoting female fecundity.. P Roy Soc Lond B.

[29] Chippindale AK, Gibson JR, Rice WR (2001). Negative genetic correlation for fitness between sexes reveals ontogenetic conflict in *Drosophila*.. P Natl Acad Sci USA.

[30] Gibson JR, Chippindale AK, Rice WR (2002). The X chromosome is a hot spot for sexually antagonistic fitness variation.. P Roy Soc Lond B.

[31] Rice WR (1998). Male fitness increases when females are eliminated from gene pool: Implications for the Y chromosome.. P Natl Acad Sci USA.

[32] Greenspan RJ, Ferveur J-F (2000). Courtship in Drosophila.. Annu Rev Genet.

[33] Talyn BC, Dowse HB (2004). The role of courtship song in sexual selection and species recognition by female *Drosophila melanogaster.*. Anim Behav.

[34] Savarit F, Sureau G, Cobb M, Ferveur JF (1999). Genetic elimination of known pheromones reveals the fundamental chemical bases of mating and isolation in *Drosophila*.. P Natl Acad Sci USA.

[35] Blows MW (2002). Interaction between natural and sexual selection during the evolution of mate recognition.. P Roy Soc Lond B.

[36] Howard RW, Jackson LL, Banse H, Blows MW (2003). Cuticular hydrocarbons of *Drosophila birchii* and *D-serrata*: Identification and role in mate choice in *D-serrata*.. J Chem Ecol.

[37] Tompkins L, Hall JC, Hall LM (1980). Courtship-stimulating volatile compounds from normal and mutant *Drosophila.*. J Insect Physiol.

[38] Grillet M, Dartevelle L, Ferveur J-F (2006). A *Drosophila* male pheromone affects female sexual receptivity.. P Roy Soc Lond B.

[39] Gleason JM, Nuzdhdin SV, Ritchie MG (2002). Quantitative trait loci affecting a courtship signal in *Drosophila melanogaster*.. Heredity.

[40] Moehring AJ, Mackay TFC (2004). The Quantitative Genetic Basis of Male Mating Behavior in *Drosophila melanogaster*.. Genetics.

[41] Chippindale AK, Leroi AM, Kim SB, Rose MR (1993). Phenotypic plasticity and selection in Drosophila life-history evolution. 1. Nutrition and the cost of reproduction.. J Evol Biol.

[42] Stewart AD, Morrow EH, Rice WR (2005). Assessing putative interlocus sexual conflict in *Drosophila melanogaster* using experimental evolution.. P Roy Soc Lond B.

[43] Chippindale AK, Leroi AM, Saing H, Borash DJ, Rose MR (1997). Phenotypic plasticity and selection in Drosophila life history evolution. 2. Diet, mates and the cost of reproduction.. J Evol Biol.

[44] Long TAF, Rice WR (2007). Adult locomotory activity mediates intralocus sexual conflict in a laboratory-adapted population of *Drosophila melanogaster.*. P Roy Soc Lond B.

[45] Chippindale AK, Rice WR (2001). Y chromosome polymorphism is a strong determinant of male fitness in *Drosophila melanogaster*.. P Natl Acad Sci USA.

[46] Drapeau MD, Cyran SA, Viering MM, Geyer PK, Long AD (2006). A *cis*-regulatory Sequence Within the *yellow* Locus of *Drosophila melanogaster* Required for Normal Male Mating Success.. Genetics.

[47] Steele RH, Partridge L (1988). A courtship advantage for small males in *Drosophila subobscura*.. Anim Behav.

[48] Crompton B, Thomason JC, McLachlan A (2003). Mating in a viscous universe: the race is to the agile, not to the swift.. P Roy Soc Lond B.

[49] Raihani G, Szekely T, Serrano-Meneses MA, Pitra C, Goriup P (2006). The influence of sexual selection and male agility on sexual size dimorphism in bustards (Otididae).. Anim Behav.

[50] Chippindale AK, Ngo AL, Rose MR (2003). The devil in the details of life-history evolution: instability and reversal of genetic correlations during selection on *Drosophila* development.. J Genet.

[51] Shakarad M, Prasad NG, Rajamani M, Joshi A (2001). Evolution of faster development does not lead to greater fluctuating asymmetry of sternopleural bristle number in Drosophila.. J Genet.

[52] Møller AP, Thornhill R (1998). Bilateral symmetry and sexual selection: A meta-analysis.. Am Nat.

[53] Markow TA (1987). Behavioral and sensory basis of courtship success in *Drosophila melanogaster.*. P Natl Acad Sci USA.

[54] Polak M, Taylor PW (2007). A primary role for developmental instability in sexual selection.. P Roy Soc Lond B.

[55] Bourguet D (2000). Fluctuating asymmetry and fitness in *Drosophila melanogaster.*. J Evol Biol.

[56] Svetec N, Ferveur JF (2005). Social experience and pheromonal perception can change male-male interactions in *Drosophila melanogaster.*. J Exp Biol.

[57] Byrne PG, Rice WR (2005). Evidence for adaptive male mate choice in the fruit fly *Drosophila melanogaster.*. P Roy Soc Lond B.

[58] Parisi M, Nuttall R, Naiman D, Bouffard G, Malley J (2003). Paucity of genes on the Drosophila X chromosome showing male-biased expression.. Science.

[59] Rose MR, Drapeau MD, Yazdi PG, Shah KH, Moise DB (2002). Evolution of late-life mortality in Drosophila melanogaster.. Evolution.

[60] Jallon JM, David JR (1987). Varaitions in cuticular hydrocarbons among the 8 species of the *Drosophila melanogaster* subgroup.. Evolution.

[61] Foley B, Chenoweth SF, Nuzhdin SV, Blows MW (2007). Natural genetic variation in cuticular hydrocarbon expression in male and female *Drosophila melanogaster.*. Genetics.

[62] Pischedda A, Chippindale AK (2006). Intralocus sexual conflict diminishes the benefits of sexual selection.. PLoS Biology.

[63] Albert AYK, Otto SP (2005). Sexual selection can resolve sex-linked sexual antagonism.. Science.

[64] Rice WR (1984). Sex-chromosomes and the evolution of sexual dimorphism.. Evolution.

